# Bayesian Multi-Targets Strategy to Track *Apis mellifera* Movements at Colony Level

**DOI:** 10.3390/insects13020181

**Published:** 2022-02-09

**Authors:** Jordão N. Oliveira, Jônatas C. Santos, Luis O. Viteri Jumbo, Carlos H. S. Almeida, Pedro F. S. Toledo, Sarah M. Rezende, Khalid Haddi, Weyder C. Santana, Michel Bessani, Jorge A. Achcar, Eugenio E. Oliveira, Carlos D. Maciel

**Affiliations:** 1 Laboratório de Processamento de Sinais, Departamento de Engenharia Elétrica, Universidade de São Paulo, São Carlos 13566-590, SP, Brazil; jordao.oliveira@usp.br (J.N.O.J.); jonatascrs@usp.br (J.C.S.); 2 Departamento de Entomologia, Universidade Federal de Viçosa, Viçosa 36570-900, MG, Brazil; luis.viteri@mail.uft.edu.br (L.O.V.J.); carlosalmeida.ufv@gmail.com (C.H.S.A.); pedrofstoledo@gmail.com (P.F.S.T.); mirandasarah12@gmail.com (S.M.R.); weyder.santana@ufv.br (W.C.S.); 3 Programa de Pós-Graduação em Biotecnologia, Universidade Federal do Tocantins, Gurupi 77402-970, TO, Brazil; 4 Departamento de Entomologia, Universidade Federal de Lavras, Lavras 37200-900, MG, Brazil; khalid.haddi@ufla.br; 5 Department of Electrical Engineering, Federal University of Minas Gerais, Belo Horizonte 31270-901, MG, Brazil; mbessani@ufmg.br; 6 Department of Social Medicine, University of São Paulo, Ribeirão Preto 14040-900, SP, Brazil; achcar@fmrp.usp.br

**Keywords:** crowded image processing, living systems, entropy, kernel density estimations, probability distribution functions, bee contamination

## Abstract

**Simple Summary:**

The number of honey bee, *Apis mellifera* L., colonies has reduced around the globe, and one potential cause is their unintended exposure to sublethal stressors such as agricultural pesticides. The quantification of such effects at colony level is a very complex task due to the innumerable collective activities done by the individual within colonies. Here, we present a Bayesian and computational approach capable of tracking the movements of bees within colonies, which allows the comparison of the collective activities of colonies that received bees previously exposed to uncontaminated diets or to diets containing sublethal concentrations of an agricultural pesticide (a commercial formulation containing the synthetic fungicides thiophanate-methyl and chlorothalonil). Our Bayesian tracking technique proved successful and superior to comparable algorithms, allowing the estimation of dynamical parameters such as entropy and kinetic energy. Our efforts demonstrated that fungicide-contaminated colonies behaved differently from uncontaminated colonies, as the former exhibited anticipated collective activities in peripheral hive areas and had reduced swarm entropy and kinetic energies. Such findings may facilitate the electronic monitoring of potential unintended effects in social pollinators, at colony level, mediated by environmental stressors (e.g., pesticides, electromagnetic fields, noise, and light intensities) alone or in combination.

**Abstract:**

Interactive movements of bees facilitate the division and organization of collective tasks, notably when they need to face internal or external environmental challenges. Here, we present a Bayesian and computational approach to track the movement of several honey bee, *Apis mellifera*, workers at colony level. We applied algorithms that combined tracking and Kernel Density Estimation (KDE), allowing measurements of entropy and Probability Distribution Function (PDF) of the motion of tracked organisms. We placed approximately 200 recently emerged and labeled bees inside an experimental colony, which consists of a mated queen, approximately 1000 bees, and a naturally occurring beehive background. Before release, labeled bees were fed for one hour with uncontaminated diets or diets containing a commercial mixture of synthetic fungicides (thiophanate-methyl and chlorothalonil). The colonies were filmed (12 min) at the 1st hour, 5th and 10th days after the bees’ release. Our results revealed that the algorithm tracked the labeled bees with great accuracy. Pesticide-contaminated colonies showed anticipated collective activities in peripheral hive areas, far from the brood area, and exhibited reduced swarm entropy and energy values when compared to uncontaminated colonies. Collectively, our approach opens novel possibilities to quantify and predict potential alterations mediated by pollutants (e.g., pesticides) at the bee colony-level.

## 1. Introduction

Bees play relevant roles in the pollination of innumerable wild plants and crop fields, which have been estimated to generate 153 billion euros globally [1]. Honey bee populations, however, are in decline worldwide [2,3,4], and its cause is not yet fully understood, but environmental pollutants such as agricultural pesticides have been suggested as one of the leading causes of this phenomenon [5,6,7,8,9]. The pesticide-mediated problems go beyond the direct lethality of these compounds, as the potential sublethal contamination of the hive individuals has been shown to alter the visual perceptions, learning, locomotion, and foraging activities of individuals [5,6,10,11]. However, how this sublethal contamination affects colony dynamics has not received adequate attention, possibly due to the challenges associated with such measures [10,12,13].

A powerful tool for analyzing collective dynamics of animal behaviors is the utilization of video tracking [14,15,16,17]. This technique is under constant development as it has applicability in a wide range of fields [18]. Due to its lower cost and the relevant advances in computational power, video tracking has been targeted by sophisticated techniques in image processing [19,20,21,22]. However, it is still a challenge to solve complex problems, such as tracking similar objects near each other [23,24], especially under heavy background noise [18,23]. Using deep neural networks has provided significant contributions [25], but these approaches are still computationally expensive, hindering their widespread application [26]. Recent approaches to object tracking (e.g., hierarchical learned features for tracking and cognitive vision) present similar difficulties as algorithms and are limited to tracking only a few objects at the same time [27,28].

Labeling methods provide efficient performance for distinguishing between objects during multi-target tracking [29,30]. The primary challenge of the multi-target tracking algorithm is the non-swapping of the labels when two objects (for example, animals being tracked) intersect. All of these multi-target video tracking challenges occur within natural colonies of social insects such as honey bees, *Apis mellifera*, as honey bee colonies are densely composed of individuals that exhibit similarities in their form and locomotion [31]. The quantitative understanding of worker behaviors within the honey bee colonies (e.g., swarm entropy [32], kinetic energy [33,34], spatial distribution, and collective activities [35]), however, is useful to recognize colony health, which can be used as indirect evidence of potential contamination by environmental pollutants.

Here, we propose a Bayesian multi-target strategy that addresses the above constraints and establishes the theoretical framework to track the movement of several *A. mellifera* individuals at colony level. We automatically monitored the movement of *A. mellifera* individuals within natural colonies. The monitored individuals were first fed with diets containing sublethal concentrations of agricultural pesticides (i.e., a commercial formulation containing the synthetic fungicides thiophanate-methyl and chlorothalonil) commonly used to control pathogens in Brazilian fields of melon, *Cucumis melo* L. [12,36,37], which is an agricultural crop heavily dependent on bee-mediated pollination.

## 2. Materials and Methods

**Figure 1:**
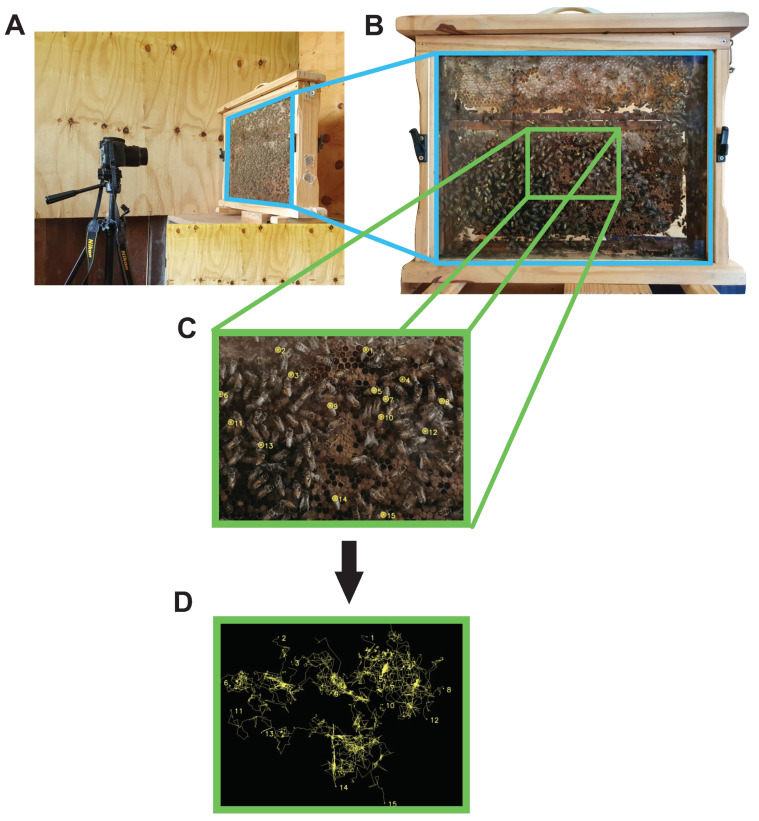
Representation of the video recording setup. An observation beehive (wooden frame with 52.5 cm x 43.5 cm) covered on the laterally with plate glass allowed the video recording of the colony. A lateral view (**A**) and a frontal view (**B**) of the experimental setup are presented. Marked workers were detected using a background subtraction algorithm and color picking (**C**). By using a Bayesian multi-target strategy, we tracked the trajectories traveled by labeled workers (either pesticide-unexposed or sublethally exposed to commercial fungicide formulation) (**D**).

In this section, we describe the experimental setup, video recording, and approaches for the preprocessing of the video frames. The algorithms for detecting and tracking each labeled individual bee are also presented. A comprehensive overview of the recording techniques and tracking is illustrated in Figure 1. The methods employed to analyze bee behavior through movement and location patterns are described.

### 2.1. Bees’ Exposure and Experimental Setup

Recently emerged (<24 h old) *A. mellifera* bees were selected from the four colonies in the experimental apiary of the Universidade Federal de Viçosa (UFV, Viçosa, MG, Brazil) and fed upon a saccharose (50%, *w*/*v*) solution that was either uncontaminated (control treatment) or had a sublethal concentration ($4.35\times 10^{-4}$ g i.a./mL) of a commercial formulation containing the synthetic fungicides thiophanate-methyl and chlorothalonil as previously described in [6]. The bees were housed in controlled environments comparable to those seen in a colony (34 ± 2 °C and 70 ± 10% relative humidity) and were kept in the dark until the experiments were completed. Afterwards, they were placed inside the observation colonies (for detail, see Figure 1A,B). We labeled the uncontaminated and pesticide-contaminated bees with different colors for easy identification. The mesonotum of each worker bee was tagged with different colors according to the contaminated and uncontaminated groups. We released 200 labeled bees in each hive that already had approximately 800 unexposed and unmarked bees.

After being reintroduced to their colonies, the bees’ activities were recorded on the 1st hour, 5th and 10th days. The initial observation periods (1st and 5th days) were defined considering the possible effect of the fungicide on behavior (e.g., walking and stopping [6]), possible mortality by manipulation, and rejection with the removal of the introduced workers from the experimental beehive due to hygienic behavior. At 10 days of age in natural conditions, workers start caring for the brood as nurse workers in the central area of the comb (task location) (for a review see in [38]). In this way, it was possible to verify the effect of the fungicide on the behavior of the workers over time within the nest. The recordings were performed using a CCD (charge-coupled device) camera (ViewPoint LifeSciences, Montreal, QC, Canada). We used four hives for each treatment, and due to the amount of data and the better technical conditions for the recordings, the hives were recorded in two periods (9:00 a.m. and 5:00 p.m.) in order to cover the moments when there are external activities occurring in the experimental colonies under natural conditions. Recording took place for 12 min in each hive at each period of the day, and all recorded videos and images were in Full HD (1920 × 1080, at 30 frames/s). The dataset comprised 48 videos (2 videos each day, for four nuclei per treatment, two treatments, and three evaluation days).

### 2.2. Image Preprocessing

The analysis of the video was performed with the extraction of its frames. A 3 frames/second rate was used in order to save computational time without losing relevant data of the bees’ activities. After that, each frame was analyzed in order to detect moving objects in the video and identify colored marks on the bees. To accomplish that, an algorithm of background subtraction [39,40] was performed, followed by a selection of colors in a previously determined range of RGB (Red-Green-Blue) values of each pixel.

The background subtraction algorithm estimated a probabilistic distribution for each pixel of the video based on a Gaussian mixture model (GMM) or k-nearest neighbors (k-NN) model [41,42]. Based on the estimated probabilistic values, the pixels of each frame of the video should be classified as background or foreground, allowing the exclusion of background information. Here, we used a k-nearest neighbors approach. After the background subtraction, each pixel was segregated according to its RGB value, and if its value was in a color range sought for tracking, it became white in accordance with the markings on the bees; otherwise, this pixel was made black.

With the objects defined as the bees’ marks, their coordinates could be evaluated by calculating their center of mass [43]. This approach is exemplified in Figure 1C. After that, each processed frame was analyzed by the algorithm from top to bottom and left to right, and each swarm object (marked workers) on the comb was labeled in order of occurrence. Because the bees are always moving, their identities (or labels) vary with each frame. If an element is labeled A in the first frame, it may be labeled B in the next frame due to its mobility. The labels of the tracked objects are reversed, which must be adjusted in order to retrieve their trajectory. The initial label must be preserved until the end of the video stream. However, even when the radius of displacement of the items is restricted, there may be more than one feasible location in the following moment due to the closeness of other bees’ positions. Similarly to that, many bees were briefly hidden behind other bees or outside of the camera capture field. Those are widely described problems in video tracking theory [23]. To solve the issues presented above, Bayesian inference was used in such a way that the next right point of the trajectory is estimated to maximize the likelihood of the preceding information, yielding a mensuration for the variable, given the prior information about the path traveled by the individuals. Then, the coordinates previously obtained were used to feed a Bayesian algorithm for tracking.

### 2.3. Bayesian Tracking

With the bees’ positions saved, the next step is to determine their correct trajectory. We are confronted with two issues here: selecting the appropriate location and the overlapping issue. The latter occurs because certain bees are overlapping with their partners and so are not visible for a period of time. To overcome this limitation, it is assumed that the bee does not move while being covered by others, thus retaining their locations. Visual investigation corroborated this empirically.

It was determined that the next correct location of the bee’s trajectory is smooth, i.e., the angle between two consecutive points is small or close to zero. The Probability Distribution Function (PDF) for the angle is assumed to be a von Mises distribution [44,45], with μ=0 and κ=4. The inference is calculated for each bee in each frame as follows: the angle variance between the bee’s potential future position, within a radius region, and the current one is evaluated. Then, the next position is optimally calculated based on the von Mises distribution, and its parameters are updated for the following step. Mathematical formulations are presented in Section S1 of the Supplementary Material.

After all iterations, a von Mises PDF is obtained for the angular displacement for each bee, and another distribution is obtained for the radius over a uniform non-informative prior. With the corrected path of each object, a Kernel Density Estimator (KDE) algorithm [41,42] creates a continuous PDF from the empirical data of angular displacement and translational displacement. More mathematical details are presented in Section S1 of the Supplementary Material.

For the real case study, the algorithm was executed on the experimental dataset of the bees previously described. The time for processing a video of approximately 12 min was ~60 min. The algorithms were developed using OpenCV 10 with Python 3.7.11.

#### Synthetic Video Simulation

As traditional databases could not offer material for the algorithm’s quality assessments, they were evaluated in a particle swarm simulation. During 500 s, a succession of frames with several dimensions and 200, 300, and 600 randomly dispersed circular objects traveling across the region was created computationally to evaluate algorithm quality and the impact of the radius on the performance.

The simulation behaves exactly like the marked bees from the previous section: the objects were chosen to be in a set of frames with the same dimensions as a Full HD video, i.e., 1080 pixels in the vertical direction and 1920 pixels in the horizontal direction, because the goal is to apply the method to real-life particle swarm videos. Complementary information about the simulation and its results are presented in Section S2 of Supplementary Material.

### 2.4. Dynamical Evaluation

Based on the acquired PDF for angle and radius, the entropy of the movement of the contaminated bees on different days may be calculated to evaluate bee dynamics. Given that the intended function of agrochemicals is to alter the metabolism of insects [33,34], this entropy should decrease with time. However, it is worthwhile to register that the entropy being assessed here is not the thermodynamical entropy, which should increase for polluted bees as they die and thus tend to reach equilibrium with the environment [46]. The entropy measured at the molecular scale certainly would grow [47].

In this work, the Shannon entropy [48] is used to evaluate the randomness of the tracked item and thus infer about their movement in space. According to the traditional view, an event’s entropy indicates how much information is required to represent the complete space state. In reality, this means that the higher the entropy, the less predictable the event, while occurrences with zero entropy have no uncertainty [49]. As it may be understood as a measurement of the randomness of the variable, the entropy of a system can give insight into the nature of the evaluated variables [48,50]. In the context of motion analysis, a greater entropy suggests that the object’s route is less predictable. For each bee, the entropy was determined using the algebraic formulation of the PDF acquired by KDE (expressed in Section S1 of the Supplementary Material).

Another measure to evaluate the dynamics of the bees is Kinetic Energy (KE). KE is a measure of how many tracked objects are moving, and it is given by the equation $\mathrm{KE}=mv^{2}/2$, where *m* is the object mass, and *v* is its velocity. Therefore, assuming all bees have the same mass, and as KE is proportional to the velocity squared, variations of energy might be represented just by $v^{2}$. Decreased energy suggests lower entropy as movement becomes less random and can provide crucial insights into a variety of real-world systems. A lower KE suggests that a bee spends more time not moving and, combined with entropy, indicates improper insect behavior [33,34].

Further details and mathematical formulation for both dynamical measures presented in this section are available in Section S3 of the Supplementary Material.

## 3. Results

### 3.1. Spatial Distribution

**Figure 2:**
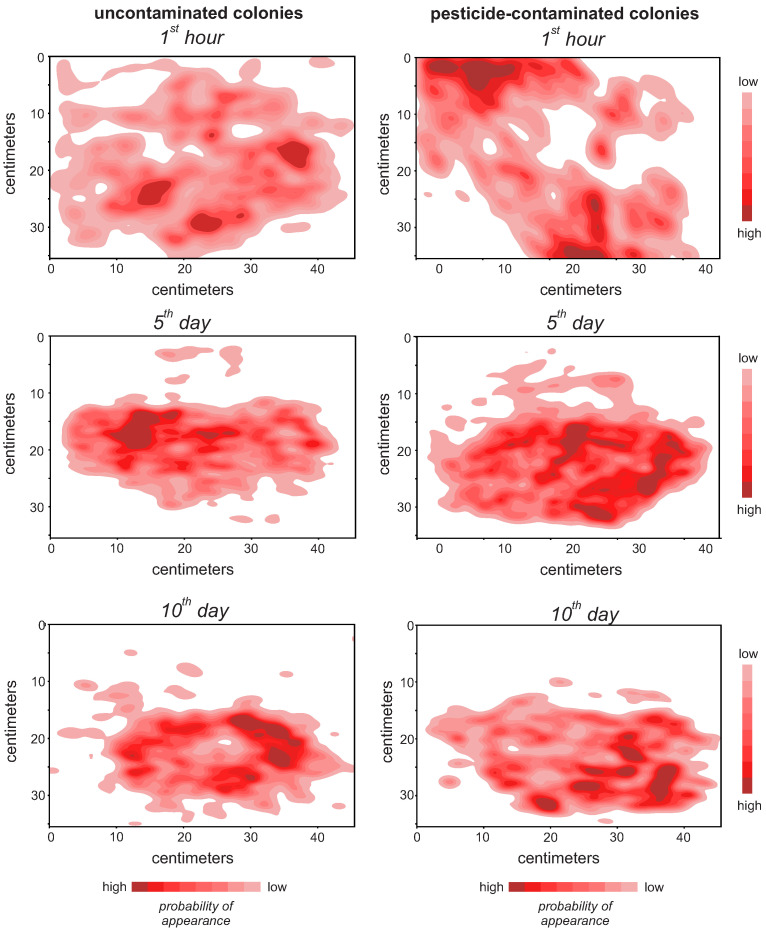
Spatial distribution of labeled *Apis mellifera* individuals at the 1st hour, 5th and 10th days after being introduced within a hive. Higher intensity color represents a higher probability of having a worker bee occupying the region. The labeled bees consisted of either pesticide-unexposed individuals (uncontaminated beehives) or individuals that were sublethally exposed to a commercial formulation containing the synthetic fungicides chlorothalonil and thiophanate-methyl (pesticide-contaminated beehives). The heatmaps indicate the number of labeled worker bee appearances in each comb region. As shown in Figure 1, the hive’s entrance is located at the lower right side of each panel representation.

The distribution of bees in the colony over time was determined by counting the number of centroids in each comb area at each frame. In order to provide intuitive analysis, a map of this distribution was generated using the Seaborn library for Python, which is presented in Figure 2. In those colonies that received fungicide-contaminated bees, there was an earlier, faster, and more intense spread of bees’ activities from the center to the boards when compared to those colonies that received only uncontaminated bees.

### 3.2. Dynamical Measurements

**Figure 3:**
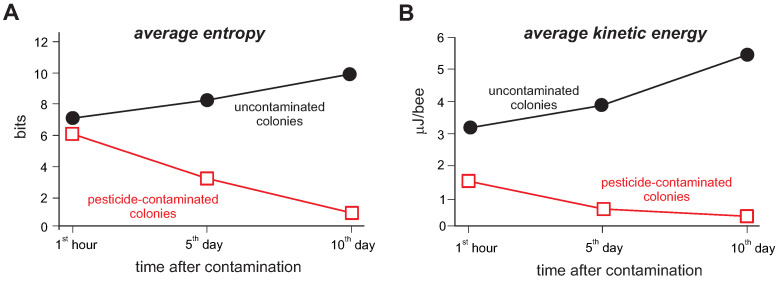
Dynamic quantification of swarm entropy (**A**) and kinetic energy (**B**) within a hive of *Apis mellifera* bees. The measurements were based on the trajectories traveled by labeled bees (i.e., either pesticide-unexposed individuals or individuals that were sublethally exposed to a commercial formulation containing the synthetic fungicides chlorothalonil and thiophanate-methyl). Each symbol represents the average value of four colonies. The measurements were conducted at the 1st hour, 5th and 10th day after the introduction of labeled bees within the colony.

**Figure 4:**
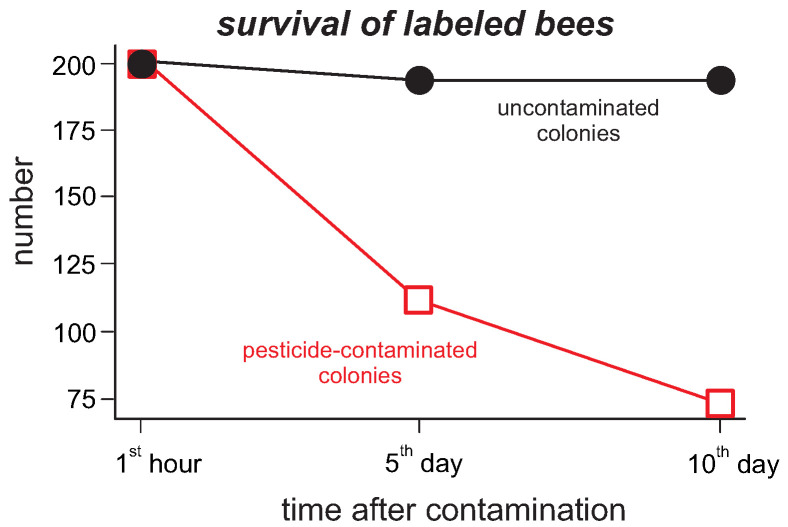
Temporal quantifications of labeled *Apis mellifera* individuals within observational hives. Each symbol represents the average value of four colonies. The measurements were conducted at the 1st hour, 5th and 10th day after the introduction of labeled bees within the hive.

The trajectories obtained from the Bayesian algorithm, through KE calculations (using the average velocity), revealed that the energy decreases from the first to the last evaluation day for beehives that received fungicide-contaminated worker bees (Figure 3A). On the other hand, it is noticeable that the KE values considerably increased in the last evaluation day for the hives that received labeled non-contaminated bees (Figure 3A). Based on the PDF values of each bee, there was a notable reduction in the entropy values over time for hives that received fungicide-contaminated labeled bees when compared to those hives that had only uncontaminated bees (Figure 3B). Our record of dead bees at the hive’s entrance revealed an increase in the number of labeled bees for the hives that received fungicide-contaminated bees, which is in accordance with the Bayesian algorithm that also recorded a smaller number of labeled bees in such fungicide-contaminated hives (Figure 4).

The Bayesian approach is sufficiently robust to determine the number of living bees each day. Other bees normally evict the dead bees from the colony. Because the nucleus has access to the outside environment, the number of bees might vary, but with a general decreasing tendency, as seen in Figure 4, which can be better explained as mortality by the agrochemical.

## 4. Discussion

Here, we present a Bayesian strategy capable of simultaneously tracking the movement of several *A. mellifera* individuals at the colony level, allowing determination of the entropy and probability distribution function (PDF) of tracked organisms. Such approaches facilitated the quantification of unintended effects mediated by agricultural pesticides (e.g., a commercial formulation containing the synthetic fungicides chlorothalonil and thiophanate-methyl) in *A. mellifera* colonies. Colonies that received pesticide sublethally-treated bees exhibited higher mortality levels, had lower swarm entropy and energy levels, and anticipated the collective activities for the peripheral hive areas, indicating a behavioral change possibly related to physiological alteration, which is suggested as a social behavioral strategy in honey bees [51,52].

Our Bayesian tracking algorithm reached great precision and performed well even with 200 labeled bees within a hive. Such a tracked number is much higher than those tracked by other software and algorithms used in biological measurements [28,53,54], maintaining an accuracy greater than 99% for the specified radius (r = 10). For instance, when the algorithms faced the image swap problems, the code had learnt the PDF of the object motions and did not make any errors in their trajectories. Another advantage of our Bayesian analysis strategy is its ability to detect the variations in the distribution, which makes the code usable for any application that tracks objects (or individuals) at variable color background possibilities, using algorithms with much fewer complexities than those presented elsewhere [27].

Despite its potentialities for measurements in dynamically evolving systems, entropy estimation via Bayesian inference is still a rather uncommon approach. Previous studies have dealt with high-quality background treatment [55] and tracking of unlabeled organisms [31,56], resulting in precise measurements. However, differently from our efforts, the other algorithms have the disadvantages of needing a low-noise image and lacking the ability to extract dynamical features of the system, as their identification is made by a convolutional neural network (a black-box model).

As previously described elsewhere [6,10,12,57,58], a commercial formulation of synthetic or natural pesticides can present risks to pollinator bees that surpass the mortality. Our Bayesian multi-target strategy clearly identified reductions in kinetic energy and entropy of hives containing individuals previously sublethally exposed to fungicides. The entropy evaluation revealed that pesticide-treated colonies had their behavior changed, with their movement becoming more predictable than before.

In general, sublethal doses of pesticides in bees can act as physiological stressors, altering metabolic responses (e.g., oxidative stress), glandular (e.g., hypopharyngeal, fat body), and bee brain (e.g., JH, biogenic amines), which alter their behavior and decrease longevity [52]. On the other hand, the distancing behavior has a social character, in which the contaminated worker bees distance themselves from the brood area, representing the most susceptible individuals in the colony, migrating earlier than expected to the peripheral areas of the comb [59,60], thus preserving colony health [52]. Despite fungicides seeming to represent lower acute toxicity, recent studies have shown their abilities to unbalance bee health by modifying quercetin-dependent detoxification [57,58,61], which can detrimentally affect mitochondrial regeneration and ATP production. Although further investigations are needed before drawing firm conclusions, it is worth noting that the disruption of quercetin-mediated detoxification has been proposed as one of the main causes for lifespan reductions in honey bees [61,62].

## 5. Conclusions

Our Bayesian tracking technique proved successful and superior to comparable ones in that it allowed for the estimation of dynamical parameters such as entropy and kinetic energy. Our technique can also convert past information about the system into mathematical equations and learn patterns whenever fresh data are added, which are two major benefits compared with traditional inferences. These qualities enabled the algorithm to detect and repair mistakes during processing, as well as forecast pathways and distributions in other scenarios of color segmentation, making the tracking more adaptable. Finally, the findings described here also contribute to getting a better understanding of potential unintended effects mediated by environmental stressors (e.g., pesticides, electromagnetic fields, noise, and light intensities) alone or in combination in pollinator bees at colony levels.

## Supplementary Materials

Further details and mathematical formulations for the presented method are available online at https://www.mdpi.com/article/10.3390/insects13020181/s1.
