# Supplementary material for: Bayesian Multi-Targets Strategy to Track Apis mellifera Movements at Colony Level
Source: Insects. 2022 Feb 9;13(2):181. doi: 10.3390/insects13020181 (PMC8875577; doi:10.3390/insects13020181)
Supplement: Supplementary file 1 [file insects-13-00181-s001.zip › insects-1523271-supplementary materials.pdf]

Bayesian multi-targets strategy to track *Apis mellifera* movements at colony level

(Supplementary Material)

Jordão N. Oliveira Jr.[1], Jônatas C. Santos[1], Luis O. Viteri Jumbo[2,3], Carlos H. S. Almeida[2], Pedro F. S. Toledo[2], Sarah M. Rezende[2], Khalid Haddi[4], Weyder C. Santana[2], Michel Bessani[5], Jorge A. Achcar[6], Eugenio E. Oliveira[2] and Carlos D. Maciel[1]

[1]Departamento de Engenharia Elétrica, Universidade de São Paulo, São Carlos, SP, 13566-590, Brazil.
[2]Departamento de Entomologia, Universidade Federal de Viçosa, Viçosa, MG, 36570-900, Brazil.
[3]Programa de Pós-graduação em Biotecnologia, Universidade Federal do Tocantins, Gurupi, TO, 77402-970, Brazil.
[4]Departamento de Entomologia, Universidade Federal de Lavras, Lavras, MG, 37200-900, Brazil.
[5]Department of Electrical Engineering, Federal University of Minas Gerais, Belo Horizonte, MG, 31270-901, Brazil.
[6]Department of Social Medicine, University of São Paulo, Ribeirão Preto, SP, 14040-900, Brazil.

1 Bayesian Tracking

1.1 Labelling Process, Bayesian inference and Maximum a Posterior (MAP)

The movement of the objects to be tracked on each frame was realised in polar coordinates with radius (r), and angle (θ); both were assumed to be random variables. For the correct labelling of each object movement, it was

used Bayesian inference to indicate the most probable displacement of marked bees on each frame. This method uses Bayes' theorem (Equation S1) to update the probability of a hypothesis as more information becomes available.

$$P(A \mid B) = \frac{P(B \mid A)P(A)}{P(B)}. \tag{S1}$$

Here, $P(A)$ and $P(B)$ are the probabilities of observing the events A and B independently; $P(A \mid B)$ is the probability of the event A, given that B happened.

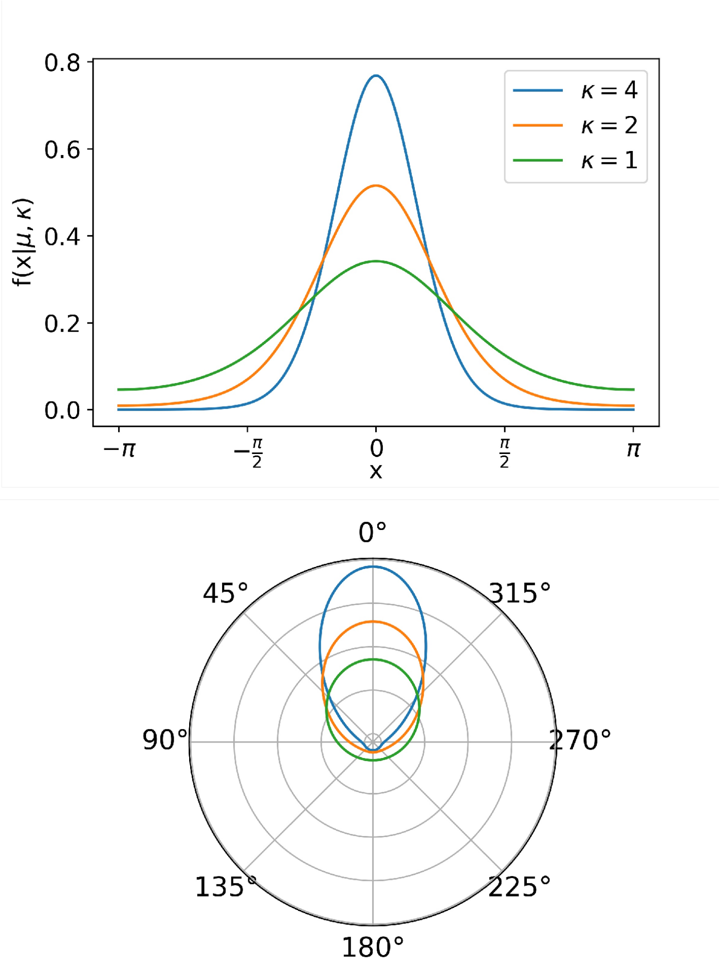

Figure S1 Comparison of the von Mises PDF for $\mu = 0$ and different values of κ. (a) If the angle variation is smooth, a good prior for the variable is $\kappa = 4$. The Bayesian algorithm will constantly update the PDF, and at the end of the process, it should give rise to the best von Mises that estimates the angular behaviour for each object being tracked. (b) Polar distribution of the probability for each value of κ.

In many applications, the event B is fixed, and it represents the impact on our belief in the occurrence of events A. In this case, the denominator $P(B)$, is fixed; what we want to vary is A. Bayes' theorem then shows that the posterior probabilities are proportional to the numerator: $P(A \mid B) \propto P(B \mid A) \cdot P(A)$. Also, the Bayes' Theorem is valid to Probability Distribution Function (PDF) and is stated as follows: let $f(x \mid \alpha)$ be the PDF of a random variable X (here, r or θ) with parameter α and samples x. Let $\pi(\alpha)$ be the prior PDF for α. Then, the posterior distribution for α given X, $\pi(\alpha)$, is defined as:

$$\pi(\alpha \mid x) = \frac{f(x \mid \alpha)\pi(\alpha)}{\int f(x \mid \alpha)\pi(\alpha)dx}. \tag{S2}$$

The estimation of the parameter α (that is not observed) can be done via the Maximum a Posterior (MAP) method. Given the observed values of X that generates a PDF $f(x \mid \alpha)$, the MAP is the maximum value of the posterior function:

$$\hat{\alpha}_{\mathrm{MAP}}(x) = \arg\max_{\theta} \pi(\alpha \mid x), \tag{S3}$$

and $\pi(\alpha \mid x)$ is calculated using Equation S2.

There were used polar coordinates to modelling the movement of the particles. For the swept angle θ, the natural choice as prior was the von Mises distribution [45, 46], since it is the circular analogue of the normal distribution. It is defined as follows:

$$f(x \mid \mu, \kappa) = \frac{e^{\kappa \cos(x-\mu)}}{2\pi I_0(\kappa)}, \tag{S4}$$

where $I_0(x) = \frac{1}{\pi}\int_0^{\pi} e^{\cos(x\cos(\theta))}d\theta$ is the modified Bessel function of order 0. In Equation S4, μ is the mean of the distribution and κ defines the curve's format (Figure S1).

For the radius, a uniform non-informative prior was chose. The algorithm has its maximum efficiency when $r \sim \lim_{n\to 0} U(0, n)$. In each iteration, a new vector $v_i = (r_i \angle \theta_i)$ will be generated (and the null vector is possible) so that the j-th particle, after all iterations, have a trajectory given by $\bigcup_{i=1}^{\infty} v_i$

1.2 Kernel Density Estimation (KDE)

This mathematical method was used to generate the continuous PDF of the empirical distributions for the angles and radius travelled by each bee, allowing for the entropy calculation. The KDE is a non-parametric algorithm to find a continuous better estimate of PDF from discretely observed data. As can be found in Terrell et al. [43], the estimated PDF is given by:

$$\hat{f}(\mathbf{y}) = \frac{1}{nh^d}\sum_{i=1}^{n} K\left(\frac{x_i - \mathbf{y}}{h}\right) \tag{S5}$$

In equation S5, $\mathbf{y}$ is the variable of the domain, x_i are the observed data, K is an interpolation function generally chosen as a Gaussian. $K : R^d \to R$ is a

function centred in 0 that integrates to 1 , and h is a smoothing parameter that would usually tend to 0 as the sample size n tends to infinite. The Loftsgaarden et al. [42] algorithm implementation for the KDE will be used in this paper, which is based in the k-nearest neighbours algorithm. It consists in, for any point of a set, its class will be assigned comparing the classes of its k nearest points. In a ball centred at x, the ratio of observations is k/n, and the KDE is given by:

$$\hat{f}(\mathbf{y}) = \frac{k}{nV_d h_k(\mathbf{y})^d}. \tag{S6}$$

In equation S6, $h_k\left(\mathbf{y}^d\right)$ is the Euclidean distance between $\mathbf{y}$ and the k-th nearest neighbour, and V_d is the volume of the unit sphere centered in the origin of the R^d space. If K is an uniform density on the unit d-sphere S_d; then

$$\hat{f}(\mathbf{y}) = \frac{k}{nh_k(\mathbf{y})^d} \sum_{i=1}^{n} K\left(\frac{x_i - \mathbf{y}}{h_k(\mathbf{y})}\right). \tag{S7}$$

1.3 Bayesian inference and dynamical evaluation

With the position of the bees stored, the next step is to trace their correct trajectory. Here we are faced with two problems: choosing the right place and the overlapping issue. The latter is caused by the fact that some bees are overlapped by its partners and therefore are not visible for some time. To surpass this hindrance, it is assumed that the bee does not move while overlapped by others, maintaining their positions. This was empirically confirmed via visual inspection.

It was adopted that the right next position of the bee's trajectory tends to be smooth. It corresponds to a prior for the angle between two consecutive points is small or close to zero. The PDF for the angle is assumed to be a von Mises with $\mu = 0$ and $\kappa = 4$ (Figure 3). The inference is calculated for each bee in each frame as follows: it is evaluated the angle variation between the possible future positions of the bee and the current one. Then, the next position is chosen to be the one to maximise the value of $f(x \mid \mu, \kappa)$. The mean μ is updated as the weighted average between the current mean and the angular variation are discovered, and κ is modelled as $\pi(\kappa) \propto U(0, 20)$. Then, Equation S4 is applied to the prior to generate the posterior for κ.

At the end of the process, it is generated a von Mises PDF for the angular displacement for every bee, as well as a distribution for the radius. Then, the KDE algorithm takes the empirical data of the angular displacement and the translational displacement, using the corrected path of each bee, and generates a continuous PDF for them. Finally, the MAP is calculated, with the prior assumed to be the von Mises of the last session for the angle.

In the process of returning the PDF for angle and radius, it also becomes possible to calculate the entropy of the movement of the contaminated bees in different days. Since the expected action of the agrochemicals is to change the metabolism of the insects [34, 35], this entropy should decrease with time.

2 Synthetic Video Simulation

A sequence of frames with several dimensions with 200, 300 and 600 randomly distributed circular objects moving through the area, during 500 seconds. The PDFs of the angle swept by the objects was defined as a von Mises, with $\mu = 0$ and $\kappa = 4$. The radius of movement was given initially by a non-informative Uniform distribution ($r \sim U(0, 50)$ pixels, or roughly 2.8 cm). After some tests in the algorithm, the radius was changed to 10 pixels (0.56 cm) and the sample time was reduced from five times as well. For each configuration the simulation was executed a thousand times. Then, the radius was changed to verify how it affects the quality of the tracking, and identify the problems involved.

The simulations were chosen to take place in a set of frames of same dimensions of a Full HD video (1080 pixels in the vertical direction and 1920 in the horizontal direction), since the objective is to apply the method for real life videos of particle swarms.

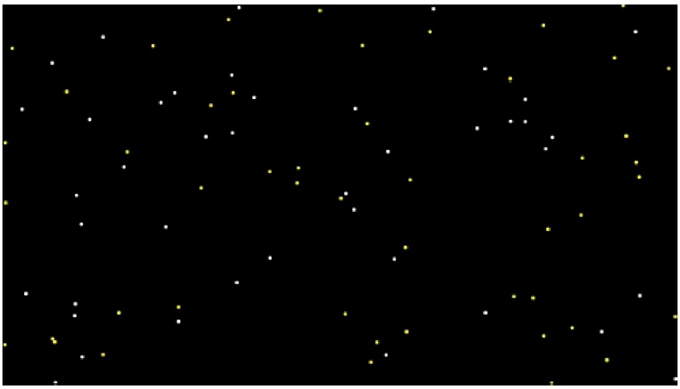

Figure S2 A frame from the video simulation. The objects generated are points with radius of 5 pixels and with colors white and yellow, and they move randomly in the background.

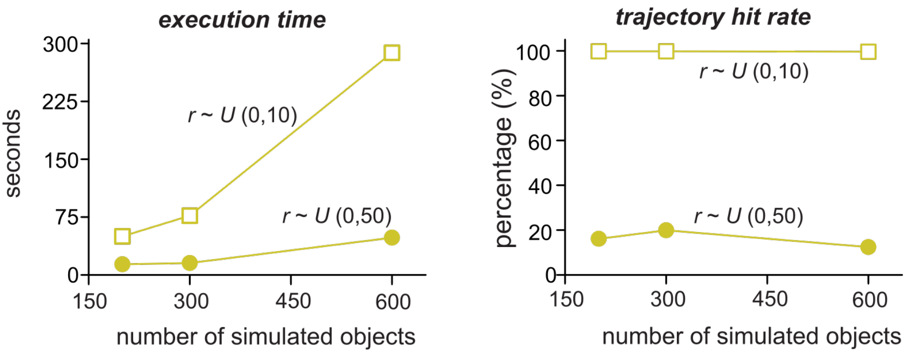

Figure S3 Efficiency of the Bayesian algorithm in the simulation, for $r \sim U(0, 50)$ and for $r \sim U(0, 10)$. Both cases with dimensions 1080x1920.

2.1 Results

An frame taken from a simulation can be seen in Figure S2 The efficiency of the Bayesian algorithm was measured in the simulation. With $r \sim U(0, 50)$, the results were fast, but hit rate was low, as can be seen in Figure S3. On the other hand, using $r \sim U(0, 10)$ results in slower execution and a greater hit rate (Figure S3). This choice for the radius was taking in account a division of 1 frame per second, and, increasing the number of frames should increase the hit rate of the algorithm, as well as the execution time. The main reason for the difference of times is that, as can be seen in Equation S2, an integral needs to be calculated each iteration, for each bee. Changing the radius increases the number of iterations.

3 Dynamical Evaluation

The Shannon entropy is used in this study to evaluate the randomness of the tracked object, and thus infer about the movement of them in space. The Shannon's entropy [49] H of an event X, with discrete PDF is given by:

$$H(X) = -\sum_{x \in X} p(x) \log_2 p(x). \tag{S8}$$

The unit of H is bits. One classical interpretation of its meaning is: the entropy of an event with n distinct outcomes (given by the variable $X = (x_1, \ldots, x_n)$) measures how much information is needed to describe the entire space state. In practice, it means that the higher the entropy, less predictable is the event, and events that are certain have zero entropy. The equivalent expression to continuous distributions is given by the differential entropy:

$$h(f) = -\int_{x \in X} f(x) \log_2 f(x) dx. \tag{S9}$$

This measurement is a generalization of the Shannon entropy, and since the PDF can be greater than 1 , even though negative entropy is difficult to attribute physical meaning. Computational computations of continuous distributions of probability inevitably go through quantization processes. Let the continuous random variable X, with distribution $f(x)$. Because, by constructing the ordered body of real numbers [41], $P(X = x) = 0$ for all x, the probability of continuous events can only be evaluated in a given interval. Therefore, any interval would need infinite bits to be fully described. However, by dividing bins of size Δ, there exists a x_i within each interval (bin) such that by the Mean Value Theorem for integrals, one can write the following approximation by Jaynes [51]:

$$H_N(X) \approx \log(N) - \log(r) + H(f). \tag{S10}$$

Since usually $N \gg r$, the Jaynes correction tries to ensure that the entropy is always positive, surpassing the difficulties of defining the meaning of the generalization of discrete entropy in the negative cases.

The entropy of a system can provide insights into the nature of the evaluated variables since it can be interpreted as a measurement of randomness of the variable [49]. In the case of motion analysis, greater entropy indicates that path travelled by the object is less predictable. A discussion of the degrees of freedom in biological systems can be found in Popovic et al. [33].

The KE is a measure of how much tracked objects are moving, and it is given by the equation KE $= mv^2/2$. Therefore, KE is proportional to the velocity squared. Lower energy indicates lower entropy, since the movement become less random and can have important insights in different real systems, as the bees in the application. A lower KE indicates that a bee spends more time not moving, and shows, together with entropy, abnormal behaviour of the insect [34, 35].
